# Bacterial Dispersal Promotes Biodegradation in Heterogeneous Systems Exposed to Osmotic Stress

**DOI:** 10.3389/fmicb.2016.01214

**Published:** 2016-08-03

**Authors:** Anja Worrich, Sara König, Thomas Banitz, Florian Centler, Karin Frank, Martin Thullner, Hauke Harms, Anja Miltner, Lukas Y. Wick, Matthias Kästner

**Affiliations:** ^1^UFZ - Helmholtz Centre for Environmental Research, Department of Environmental MicrobiologyLeipzig, Germany; ^2^UFZ - Helmholtz Centre for Environmental Research, Department of Environmental BiotechnologyLeipzig, Germany; ^3^UFZ - Helmholtz Centre for Environmental Research, Department of Ecological ModellingLeipzig, Germany; ^4^German Centre for Integrative Biodiversity Research (iDiv) Halle-Jena-LeipzigLeipzig, Germany; ^5^Institute for Environmental Systems Research, University of OsnabrückOsnabrück, Germany

**Keywords:** spatial processes, heterogeneity, biodegradation, dispersal networks, *Pseudomonas putida*, osmotic stress, diffusion, contaminants

## Abstract

Contaminant biodegradation in soils is hampered by the heterogeneous distribution of degrading communities colonizing isolated microenvironments as a result of the soil architecture. Over the last years, soil salinization was recognized as an additional problem especially in arid and semiarid ecosystems as it drastically reduces the activity and motility of bacteria. Here, we studied the importance of different spatial processes for benzoate biodegradation at an environmentally relevant range of osmotic potentials (ΔΨ_o_) using model ecosystems exhibiting a heterogeneous distribution of the soil-borne bacterium *Pseudomonas putida* KT2440. Three systematically manipulated scenarios allowed us to cover the effects of (i) substrate diffusion, (ii) substrate diffusion and autonomous bacterial dispersal, and (iii) substrate diffusion and autonomous as well as mediated bacterial dispersal along glass fiber networks mimicking fungal hyphae. To quantify the relative importance of the different spatial processes, we compared these heterogeneous scenarios to a reference value obtained for each ΔΨ_o_ by means of a quasi-optimal scenario in which degraders were *ab initio* homogeneously distributed. Substrate diffusion as the sole spatial process was insufficient to counteract the disadvantage due to spatial degrader heterogeneity at ΔΨ_o_ ranging from 0 to −1 MPa. In this scenario, only 13.8−21.3% of the quasi-optimal biodegradation performance could be achieved. In the same range of ΔΨ_o_ values, substrate diffusion in combination with bacterial dispersal allowed between 68.6 and 36.2% of the performance showing a clear downwards trend with decreasing ΔΨ_o_. At −1.5 MPa, however, this scenario performed worse than the diffusion scenario, possibly as a result of energetic disadvantages associated with flagellum synthesis and emerging requirements to exceed a critical population density to resist osmotic stress. Network-mediated bacterial dispersal kept biodegradation almost consistently high with an average of 70.7 ± 7.8%, regardless of the strength of the osmotic stress. We propose that especially fungal network-mediated bacterial dispersal is a key process to achieve high functionality of heterogeneous microbial ecosystems also at reduced osmotic potentials. Thus, mechanical stress by, for example, soil homogenization should be kept low in order to preserve fungal network integrity.

## Introduction

Bacterial degradation of contaminants prevents their persistence in the environment as well as the contamination of water resources due to leaching (Arias-Estévez et al., 2008). However, the distribution of bacterial degraders in soils shows a distinct spatial heterogeneity in both horizontal (Vinther et al., 2008) and vertical (Badawi et al., 2013) direction. For nearly a century, spatial heterogeneity was considered only a large-scale phenomenon (Dechesne et al., 2014), and microbial ecologists stuck to the “Everything is everywhere” paradigm suggesting that microorganisms are mostly cosmopolitan and almost homogeneously distributed (Cho and Tiedje, 2000). Owing to intensive work on spatial heterogeneity in soil, the endemism hypothesis for bacteria is now generally accepted (Cho and Tiedje, 2000; Fulthorpe et al., 2008) and there is increasing awareness that heterogeneity is not only a field-scale phenomenon, but similarly occurs at the micro-scale (Vieublé Gonod et al., 2006). Studies on the distribution of 2,4-dichlorophenoxyacetic acid (2,4-D) degraders in soil and soil column systems confirmed the existence of soil regions comprising several millimeters right up to a few centimeters, which are devoid of 2,4-D degrading activity (Pallud et al., 2004; Vieublé Gonod et al., 2006; Pinheiro et al., 2015). However, the majority of publications dealing with spatial heterogeneity of bacterial degraders in soils are rather descriptive, whereas the consequences of these small-scale spatial heterogeneities on biodegradation efficiency are difficult to assess and thus have hardly been evaluated so far (Dechesne et al., 2014).

Bacterial dispersal and substrate mass transfer are considered key factors for efficient biodegradation as both processes help to overcome spatial separation, thus leading to increased contact probability between bacteria and contaminants in a spatially heterogeneous environment like soil (Semple et al., 2007). Motility is a highly conserved trait among bacteria and more than two thirds of the sequenced species are motile, indicating that it provides bacteria with an essential ecological advantage (Czaban et al., 2007; Wei et al., 2011). Motile bacteria, for example, can actively disperse in the pore water and spread along dense mycelial networks formed by fungi leading to improved phenanthrene biodegradation (Wick et al., 2007).

Besides being often contaminated, soils are highly affected by salt accumulation due to low rainfall and high evapotranspiration (Rengasamy, 2006). Globally, more than 831 million hectares of land are affected by salt at levels causing osmotic stress in bacteria (Martinez-Beltran and Manzur, 2005). Recently, it was shown that the presence of mycelium-like networks improved dispersal, growth, and biodegradation under environmental stress conditions induced by lowered water potentials (Worrich et al., 2016). However, to what extent spatial processes like bacterial dispersal, network-mediated bacterial dispersal, or substrate diffusion may counteract the disadvantage due to a heterogeneous degrader distribution at different osmotic potentials remains unclear. Indeed, it is highly relevant to investigate whether and under which environmental conditions the spatial heterogeneity of degrading bacteria has to be taken into account in order to (i) improve models of microbial biodegradation in soils, (ii) predict contaminant fate in the environment, and (iii) derive strategies for risk assessment and management (Holden and Firestone, 1997; Soulas and Lagacherie, 2001).

Here, we aimed at quantifying the effect of different spatial processes (i.e., bacterial dispersal and substrate diffusion) on biodegradation efficiency at a range of environmentally relevant osmotic potentials, which are known to cause restrictions in bacterial growth and motility. We tested the hypothesis that to some extent autonomous bacterial dispersal but especially the dispersal along mycelia is crucial to counteract the disadvantages evoked by spatial degrader heterogeneity as sufficient compensation cannot be achieved by substrate diffusion alone. Furthermore, we wanted to assess whether there is a certain threshold at which not the spatial processes but rather the physiological limitations control biodegradation efficiency. In a microbial model system, we created different scenarios permitting solely substrate diffusion, or substrate diffusion and autonomous bacterial dispersal, or the two aforementioned processes plus network-mediated bacterial dispersal. A comparison of the respective scenarios to a reference scenario exhibiting a homogeneous degrader distribution allowed for a relative quantification of the biodegradation promoting effects for the different spatial processes.

## Methods

### Organisms and culture conditions

Experiments were carried out with a benzoate-degrading, GFP-tagged derivative of the soil bacterium *Pseudomonas putida* KT2440. It was cultivated in FAB minimal medium (Hansen et al., 2007) supplemented with 50 mM sodium benzoate (FAB-50, Sigma-Aldrich, Munich, Germany) at room temperature with 150 rpm rotary culture flask movement. For strain maintenance, bacteria were transferred weekly to FAB-50 plates containing 1.5% (w/v) agar and incubated at room temperature.

### Bacterial population growth kinetics under osmotic stress

For a detailed analysis of the effects of different sodium chloride concentrations on bacterial growth kinetics, we measured the increase in optical density at 600 nm in 96-well microtiter plates (OD_600_) in a plate reader (SpectraMAX 250, Molecular Devices, California U.S.) set to 25°C. The wells were filled with 200 μl FAB-50 supplemented with either 3.2, 6.4, 12.8, and 19.2 g l^−1^ of sodium chloride, which corresponds to ΔΨ_o_ of −0.25, −0.5, −1, or −1.5 MPa (Holden et al., 1997). FAB-50 medium without sodium chloride served as ΔΨ_o_ = 0 MPa control treatment. To minimize edge effects, outer wells were filled with potassium phosphate buffer (PB, 10 mM, pH 7.2) and not used for growth analysis (Johnsen et al., 2002). Cells from a liquid culture were harvested by centrifugation at 8000 g for 10 min. The pellet was washed once with PB and adjusted to an optical density of 20 with FAB-50 medium. Each well was inoculated with 2 μl using a multichannel pipette. Plates thus contained 12 replicates per osmotic stress treatment. Measurements were carried out every 30 min over a total of 86 h. Maximum specific growth rates (μ_max_) were determined by exponentially fitting the sections of highest increase in the growth curves. Maximum biomass yields were calculated from the maximum OD_600_ values of each replicate using a calibration curve for the correlation between dry biomass and OD_600_. Samples exhibiting different OD_600_ values were filtered through weighed cellulose nitrate membrane filters with a pore size of 0.22 μm (Sartorius, Goettingen, Germany). After drying at 60°C for 48 h, filters were weighed again with the increase determining dry biomass. Lag times were derived from the x-axis intercept of the straight line in the maximum slope of a non-parametric spline fit function of the “grofit”-package in R (Kahm et al., 2010; R Core Team, 2014).

### Microcosm setup

To investigate the role of the different spatial processes, four different scenarios were created. The wells of clear and sterile 24-well flat-bottom microtiter plates were filled with either 1 ml of FAB-50 minimal medium agar of 1% (w/v) to allow for diffusion but completely restrict bacterial dispersal (*D*_*dif*_) or 0.3% (w/v) to allow for bacterial dispersal and substrate diffusion (*D*_*dis*_). To test the influence of mediated bacterial dispersal (*D*_*net*_), a glass fiber net with an area weight of 14 g m^−2^ (Mühlmeier composite, Bärnau, Germany) was placed on top of 0.3% (w/v) agar (Supplementary Figure 1). The glass fiber net was cut into circular pieces of Ø 1.2 cm using a cork borer. Subsequently, the pieces were heat sterilized at 450°C for 4 h in a muffle furnace and placed in the microcosms before inoculation with bacteria. To create the reference scenario with a homogeneous cell distribution (*D*_*hom*_), wells were filled with liquid FAB-50 minimal medium. Osmotic stress was induced similarly to the growth kinetics experiment. Different amounts of sodium chloride were added to the FAB-50 liquid medium or agar prior to autoclaving. Agar and liquid medium without additional sodium chloride served as ΔΨ_o_ = 0 MPa control treatments. Before use, all agar plates were dried in a laminar flow cabinet for 5 min.

### Microcosm inoculation

**Figure 1:**
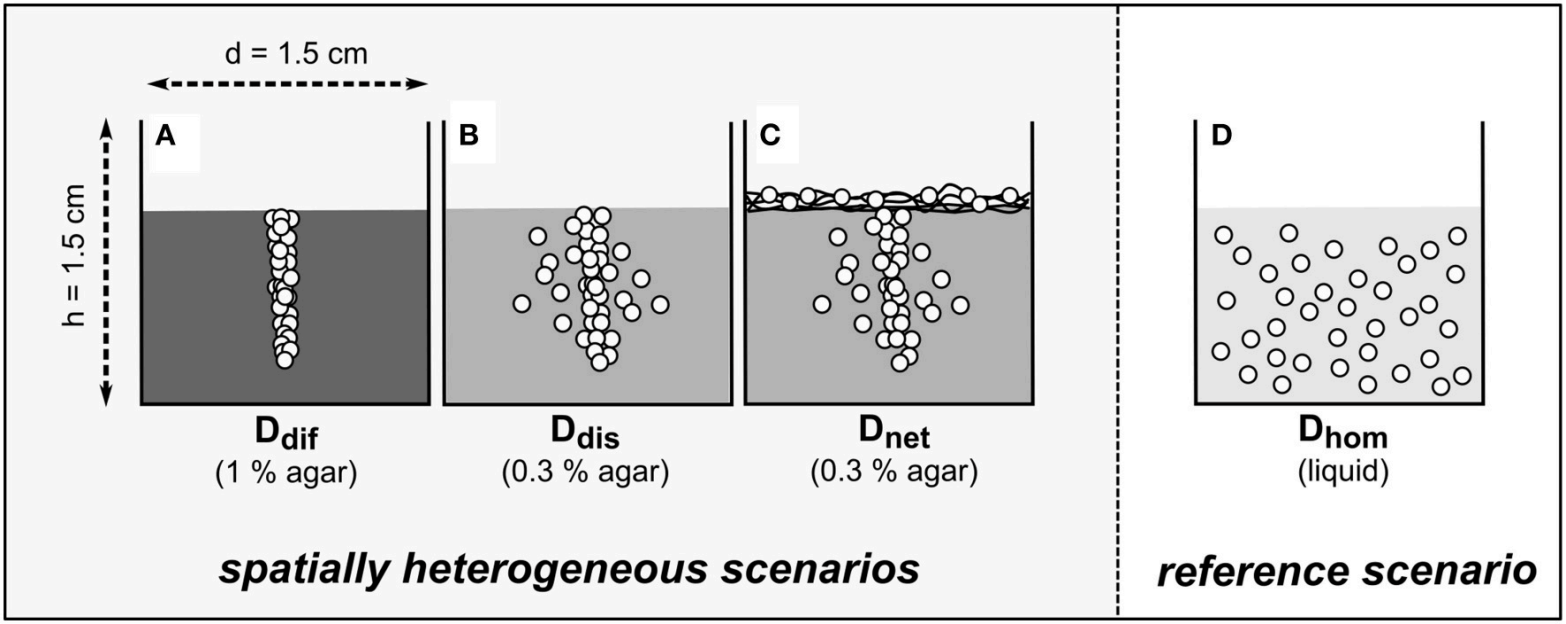
**Scheme of the model ecosystems used to assess the counteracting effects of substrate diffusion (A), substrate diffusion and autonomous bacterial dispersal (B), and substrate diffusion, autonomous bacterial dispersal, and network-mediated bacterial dispersal (C) on population growth and biodegradation in systems exhibiting a heterogeneous degrader distribution in comparison to a homogeneous reference scenario (D)**. White circles indicate the anticipated distribution of bacteria in matrices with increasing physical hindrance (indicated by shades of gray). Black lines **(C)** depict the glass fiber network used to simulate mycelial networks. In each treatment the osmotic potential ΔΨ_o_ was varied between 0 and −1.5 MPa using sodium chloride.

Cells were harvested from liquid culture by centrifugation at 8000 g for 10 min after 16 h of cultivation. The pellet was washed once with PB and adjusted to an optical density of 50. Microcosms were inoculated in the center of each well with 0.2 μl bacterial suspension [~2.4·10^6^ colony forming units (CFU)] using a microliter syringe. The needle was pricked deep into the agar (~0.8 cm; cf. Figure 1) but did not touch the bottom of the microcosms during inoculation. Abiotic control treatments were left uninoculated. To achieve a homogeneous distribution of bacterial cells in the *D*_*hom*_ scenarios, plates were shaken for 1 min at 300 rpm on a rotary shaker. Plates were incubated under static conditions in plastic containers at room temperature in the dark.

### Bacterial population growth and degradation measurement

Cell numbers and benzoate concentrations were determined after 6, 24, 30, and 48 h in replicate multiwell plates. Well contents were carefully mixed with 2 ml PB. For the agar experiments, the PB-agar slurry was subsequently transferred to a sterile Falcon tube and detachment of bacterial cells was carried out by vortexing and ultrasonication in a water bath with a frequency of 35 kHz (Sonorex Super RK 255H, Bandelin, Berlin) for 1 min. Culturable cells were analyzed as CFU. To this end, bacteria were spread on FAB-50 plates using the drop plate method as described earlier (Chen et al., 2003). Briefly, 10-fold dilution series of the supernatant were prepared directly in 96-well microtiter plates and 5 μl of consecutive dilutions were dropped on the agar plate. Plates were incubated at 25°C for 48 h. Droplets giving rise to between 5 and 30 single colonies were used to determine CFU numbers.

For benzoate measurements, the supernatant obtained from cell detachment was fixed with 4% (w/v) formaldehyde and filtered through 0.22 μm syringe filters (Carl Roth, Karlsruhe, Germany) to remove bacteria. The benzoate concentration was determined with an HPLC system equipped with a C_18_ reverse phase column (250 × 4 mm) and a Photodiode Array Detector (PDA) set at 271 nm. The system was operated at a flow rate of 1.2 ml min^−1^, 10 μl injection volume and a mobile phase consisting of 80% sodium acetate (50 mM, pH 4.5) and 20% MeOH (Warikoo et al., 1996). Benzoate had a retention time of 13.8 min under these operation conditions.

### Determination of the relative counteracting effects of bacterial dispersal and substrate diffusion at different osmotic potentials

The extent to which bacterial dispersal and substrate diffusion are able to counteract the disadvantage caused by a heterogeneous degrader distribution at different osmotic potentials and thus to maintain benzoate biodegradation was analyzed by comparing the performance of the respective scenarios (*D*_*dif*_, *D*_*dis*_, and *D*_*net*_) with the performance of the *D*_*hom*_ setup exhibiting a quasi-optimal homogeneous distribution of the degraders. We calculated the areas under the curve (AUC) for the time courses of benzoate biodegradation for the different distribution scenarios at the different osmotic potentials. The AUC serves as an aggregated measure of the temporal biodegradation performance and was calculated using the trapezoidal method in R. This numerical approximation of the time integral allows for non-equidistant time data and, thus, is not hampered by missing measurements for certain points in time. The relative counteracting effects in terms of biodegradation performance (RCE) for each scenario *D*_*i*_ in dependence of the osmotic potential ΔΨ_o_ were calculated according to:

(1) $$\begin{array}{l} {\text{RCE}}_{{D_{i},\Delta \Psi }_{\text{o}}}=\frac{{\text{AUC}}_{D_{i}}}{{\text{AUC}}_{D_{hom}}};\left(D_{1}=D_{dif};D_{2}=D_{dis};D_{3}=D_{net}\right). \end{array}$$

## Results

### Bacterial population growth kinetics under osmotic stress

**Figure 2:**
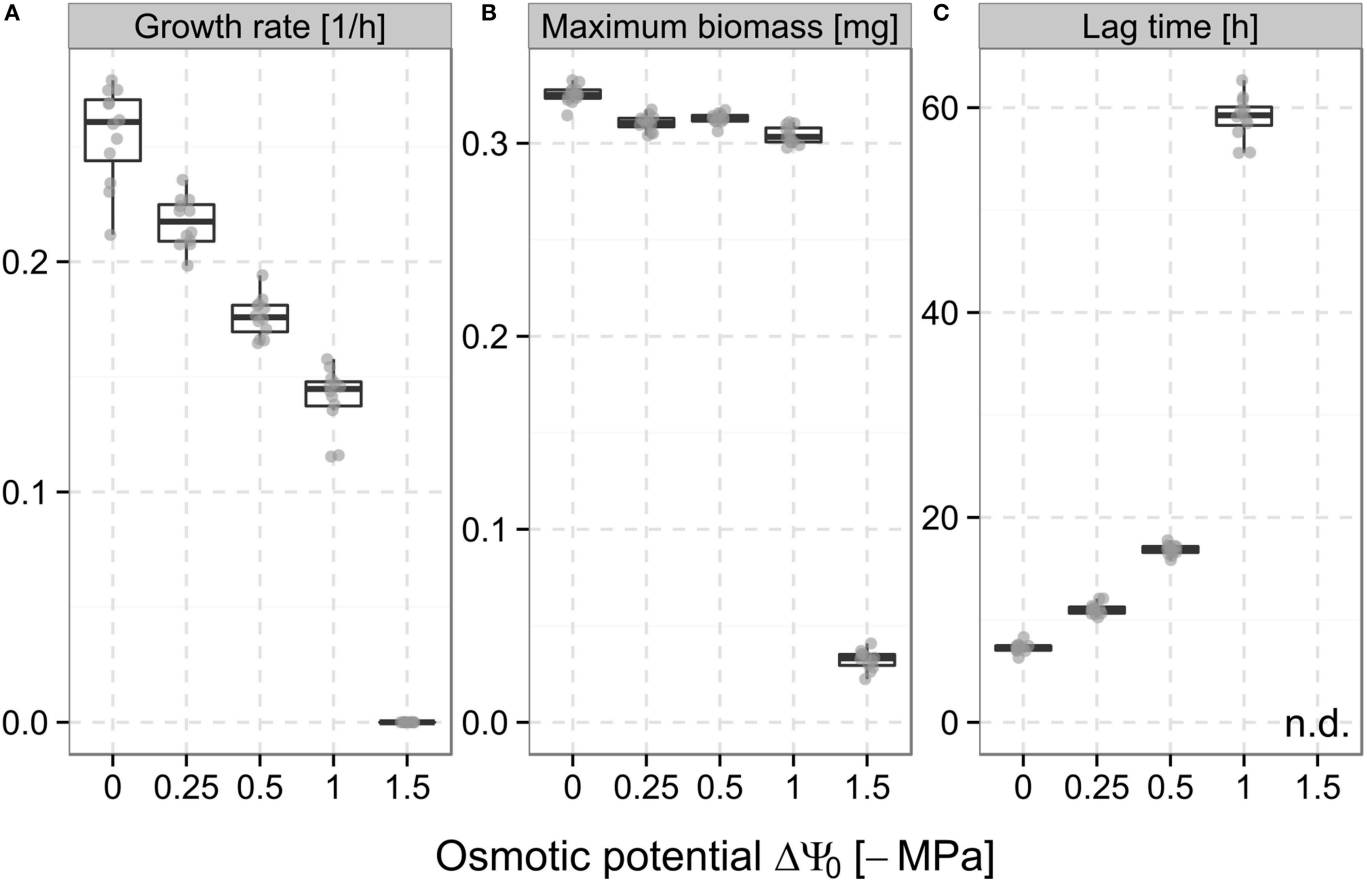
**Maximum specific growth rate (A), maximum biomass (B), and lag time (C) of ***Pseudomonas putida*** KT2440 during growth in liquid culture at the different osmotic potentials (ΔΨ_**o**_) adjusted with sodium chloride (cf. Section Bacterial Population Growth Kinetics under Osmotic Stress)**. Boxes show the medians and the interquartile range of values (between 25th and 75th percentile). Whiskers extend to values not more than 1.5-fold out of this range. Points represent the values of individual replicates (12 in total for each ΔΨ_o_).

The obtained bacterial growth curves (Supplementary Figure 2) show a distinct response to different levels of osmotic stress created by the addition of sodium chloride. With decreasing ΔΨ_o_, the maximum specific growth rates were found to gradually decrease from 0.26 h^−1^ at ΔΨ_o_ = 0 MPa to 0.14 h^−1^ at ΔΨ_o_ = −1 MPa and then dropped to 0 h^−1^ at ΔΨ_o_ = −1.5 MPa (Figure 2A). A similar behavior was observed for maximum biomass, which showed only minor changes between ΔΨ_o_ = 0 MPa and ΔΨ_o_ = −1 MPa (reduction from 0.32 to 0.3 mg) but markedly decreased to 0.03 mg at ΔΨ_o_ = −1.5 MPa, which corresponds to the inoculated biomass (Figure 2B). Lag times exhibited a different pattern as a drastic prolongation to 59 h was observed already at ΔΨ_o_ = −1 MPa compared to 7.3 h at ΔΨ_o_ = 0 MPa. At ΔΨ_o_ = −1.5 MPa, lag times could not be determined as no growth was observed within the duration of the experiments (Figure 2C).

### Effects of the spatial degrader distribution on biodegradation and population growth

**Figure 3:**
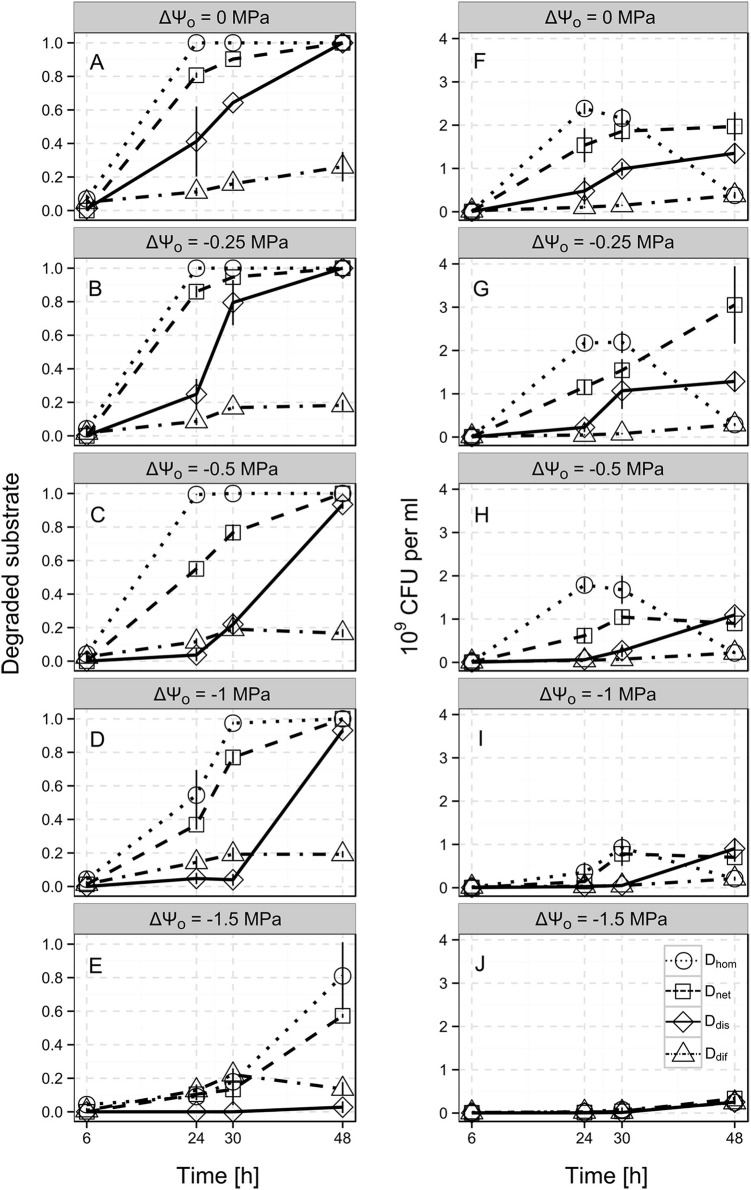
**Time-resolved benzoate biodegradation performance (A–E) and bacterial population size (F–J) of ***Pseudomonas putida*** KT2440 for the different scenarios Di at the different osmotic potentials (ΔΨ_**o**_, indicated by the subplot labels)**. Single lines show three heterogeneous scenarios allowing for either solely substrate diffusion (*D*_*dif*_; dot-dashed lines), substrate diffusion and autonomous bacterial dispersal (*D*_*dis*_; solid lines), and substrate diffusion, autonomous bacterial dispersal and network-mediated bacterial dispersal (*D*_*net*_; dashed lines) and the reference scenario with a homogeneous cell distribution (*D*_*hom*_; dotted line). Benzoate biodegradation performance and population dynamics were assessed by the relative amounts of benzoate degraded and the CFU numbers after 6, 24, 30, and 48 h. Error bars represent the standard deviation from 4 biological replicates.

The spatial distribution of the bacterial population had a drastic impact on benzoate biodegradation and bacterial population dynamics. In the *D*_*hom*_ scenario, the total amount of benzoate in the system was already degraded after 24 h, whereas in the *D*_*dif*_ scenario, only 11.1% of the total benzoate was consumed at this time point (Figure 3A, dotted and dot-dashed line). At the end of the experiment (after 48 h), 26.1% of the benzoate was consumed on average in the *D*_*dif*_ scenario (i.e., when bacteria were trapped at the inoculation point, Figure 3A, dot-dashed line). In the *D*_*dis*_ scenario, 41.2 and 64.3% of the benzoate was consumed after 24 and 30 h, whereas in the *D*_*net*_ scenario, benzoate consumption accounted for 80.7 and 90.3% at the respective time points (Figure 3A, solid and dashed line). In both scenarios, the total amount of benzoate was degraded after 48 h.

CFU numbers in the *D*_*dif*_ scenario were roughly 23-fold lower compared to the *D*_*hom*_ scenario after 24 h of incubation (1.05·10^8^ and 2.39·10^9^ CFU ml^−1^, respectively, Figure 3F). With increasing incubation time, CFU numbers in the *D*_*dif*_ scenario steadily increased to 3.8·10^8^ CFU ml^−1^ after 48 h, whereas in the D_*hom*_ scenario CFU numbers already showed a decline at 30 h (Figure 3F, dot-dashed and dotted line). At 48 h, *D*_*hom*_ and *D*_*dif*_ scenarios exhibited the same CFU number. In the *D*_*dis*_ and *D*_*net*_ scenarios, CFU numbers continuously increased to 1.35·10^9^ and 1.97·10^9^ CFU ml^−1^ at 48 h, respectively. However, in the *D*_*net*_ scenario, we consistently observed a higher CFU number at the different time points (Figure 3F, dashed and solid line).

### Impact of different spatial degrader distributions on the response of biodegradation and population growth to varying osmotic potentials

Apparently, in the *D*_*hom*_ scenario, a reduction of ΔΨ_o_ down to −0.5 MPa had no effect on biodegradation efficiency. After 24 h, the entire benzoate added to the microcosms was degraded also at ΔΨ_o_ = −0.25 MPa and ΔΨ_o_ = −0.5 MPa (Figures 3A–C, dotted line). However, lower ΔΨ_o_ of −1 MPa and −1.5 MPa led to decelerated benzoate biodegradation in the *D*_*hom*_ scenario (Figures 3D,E, dotted lines). At ΔΨ_o_ = −1 MPa, the benzoate in the *D*_*hom*_ scenarios was almost completely degraded after 30 h (97.4%), whereas at ΔΨ_o_ = −1.5 MPa still 18.9% remained in the microcosms after 48 h (Figures 3D,E). In the *D*_*dif*_ scenario, we observed only minor changes in biodegradation with decreasing osmotic potentials (Figures 3A–E, dot-dashed line). After 48 h, between 26.1% at ΔΨ_o_ = 0 MPa and 13.6% at ΔΨ_o_ = −1.5 MPa of the total benzoate was degraded.

Biodegradation in the *D*_*dis*_ scenario was markedly decelerated at lowered ΔΨ_o_. While at ΔΨ_o_ = −0.25 MPa, 24.8% of the benzoate was consumed, we did not observe any biodegradation at ΔΨ_o_ = −1.5 MPa after 24 h. At the end of the experiments (after 48 h), almost the whole amount of benzoate was degraded at ΔΨ_o_ ranging from 0 to −1 MPa, whereas at ΔΨ_o_ = −1.5 MPa still 97.2% of the benzoate was left in the microcosms. Also in the *D*_*net*_ scenario, benzoate biodegradation was decelerated at lowered ΔΨ_o_. However, the amount of degraded benzoate always exceeded the values obtained for the *D*_*dis*_ scenario and still accounted for 57.2% at ΔΨ_o_ = −1.5 MPa.

Generally, bacterial population dynamics in the *D*_*hom*_ scenario exposed to osmotic stress reflected the degradation patterns. Also here, drastic reductions in CFU numbers were observed at ΔΨ_o_ = −1 MPa with a 6.7-fold lower value compared to ΔΨ_o_ = 0 MPa after 24 h (Figures 3F,I, dotted lines). In the *D*_*dif*_ scenario, CFU numbers remained very low for all osmotic potentials (Figures 3F–J, dash-dotted lines). Moreover, the final values (after 48 h) also gradually decreased from 3.8 · 10^8^ CFU ml^−1^ for ΔΨ_o_ = 0 MPa to 2.43 ·10^8^ for ΔΨ_o_ = −1.5 MPa. For the *D*_*dis*_ and *D*_*net*_ scenarios, we observed decelerations similarly to the biodegradation patterns at decreasing ΔΨ_o_. Also final CFU numbers steadily decreased for both scenarios between ΔΨ_o_ of −0.25 and −1.5 MPa (1.35 ·10^9^ to 2.52 ·10^8^ CFU ml^−1^ for *D*_*dis*_ and 3 ·10^9^ to 3.35 ·10^8^ CFU ml^−1^ for *D*_*net*_).

### Relative counteracting effects of dispersal and diffusion at different osmotic potentials

**Figure 4:**
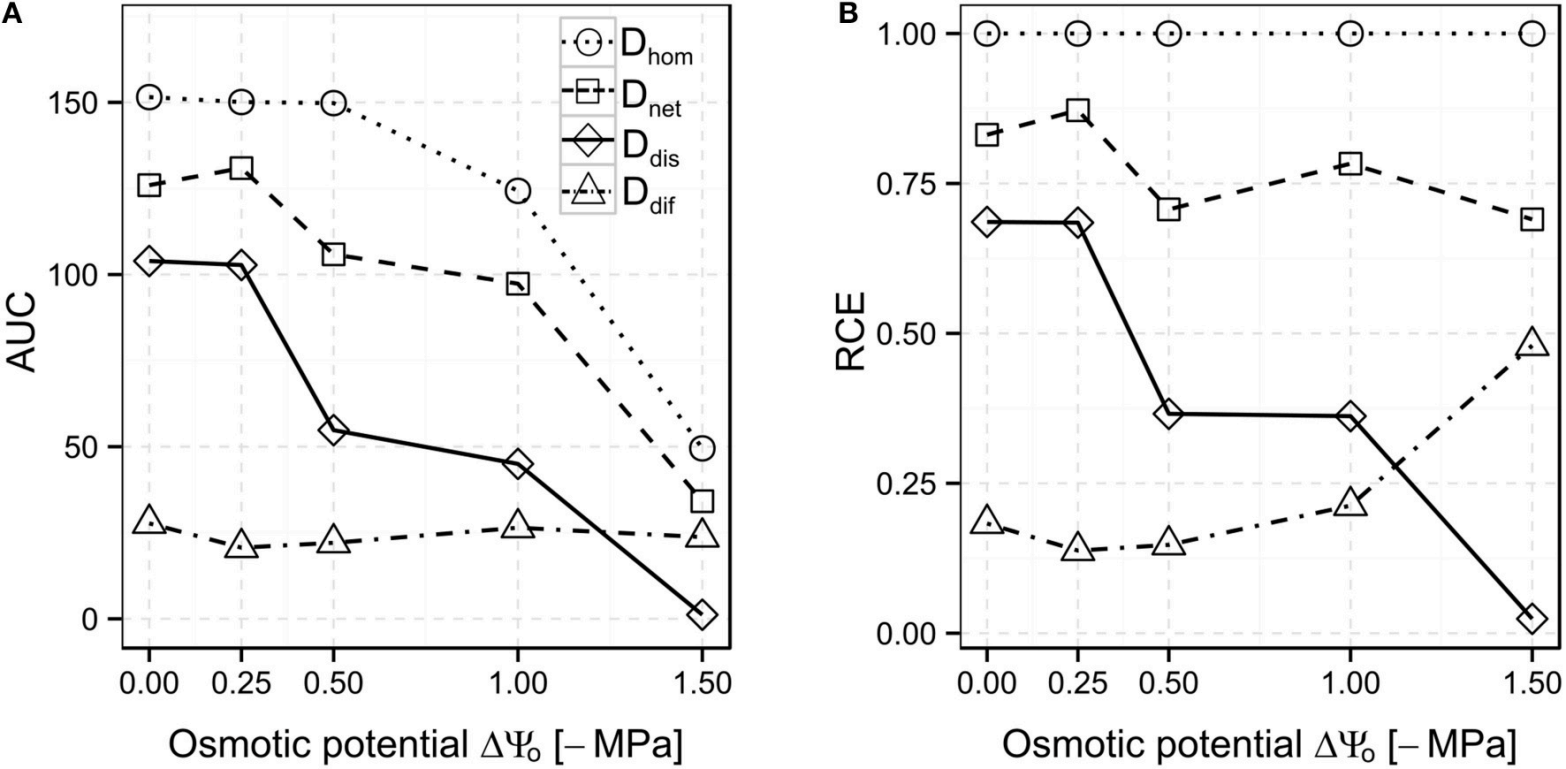
**Area under the curve (AUC; A) and Relative Counteracting Effects (RCE; B) calculated for the different scenarios ***Di*** at different ΔΨ_**o**_**. For RCE calculation, AUC values were divided by the AUC value of the reference scenario *D*_*hom*_ (cf. Equation 1).

AUC values of *D*_*hom*_, *D*_*dis*_, and *D*_*net*_ scenarios decreased with decreasing osmotic potentials, but remained almost stable at a low level in the *D*_*dif*_ scenario (24.1 ± 2.9, Figure 4A; cf. Figures 3A–E). Relative counteracting effects (RCE) were calculated by dividing the AUC values for the *D*_*dif*_, *D*_*dis*_, and *D*_*net*_ scenarios by the AUC value for the *D*_*hom*_ scenario at each osmotic potential. In the *D*_*dif*_ scenario, only 18.3% of the biodegradation performance relative to the *D*_*hom*_ scenario was achieved at ΔΨ_o_ = 0 MPa. At lowered ΔΨ_o_, RCE varied between 13.8% (ΔΨ_o_ = −0.25 MPa) and 21.3% (ΔΨ_o_ = −1 MPa), but increased to 48% at ΔΨ_o_ = −1.5 MPa (Figure 4B, dot-dashed line). Autonomous bacterial dispersal (*D*_*dis*_) could maintain biodegradation to 68.6% at ΔΨ_o_ = 0 MPa and decreased only slightly at ΔΨ_o_ = −0.25 MPa. At ΔΨ_o_ = −0.5 and −1 MPa, RCE dropped to 36.6 and 36.2%, respectively (Figure 4B, solid line). However, we consistently obtained a higher RCE in the *D*_*dis*_ compared to the *D*_*dif*_ scenario at ΔΨ_o_ from 0 to −1 MPa. The only exception occurred at ΔΨ_o_ = −1.5 MPa, where bacterial dispersal exhibited a lower RCE compared to *D*_*dis*_ (RCE of 2.4 and 48%, respectively, Figure 4B). In the *D*_*net*_ scenario, RCE was always the highest compared to the other scenarios and varied between 87.2 and 69% over the range of osmotic potentials (Figure 4B, dashed line).

## Discussion

### Importance of the spatial degrader distribution

In this study, we tested how different spatial processes can counteract a heterogeneous degrader distribution. We compared biodegradation in scenarios allowing for different spatial processes to a scenario in which degraders were homogeneously distributed to mimic optimal distribution conditions. Salinity is known to affect bacterial dispersal and thus we analyzed how the counteracting effects change with decreasing osmotic potentials and if there is some kind of threshold at which not the spatial processes but rather the physiological limitations control biodegradation efficiency. The soil bacterium *P. putida* KT2440 was chosen because of its well-characterized motility behavior (Dechesne et al., 2010b), which recently was investigated also under osmotic stress conditions (Worrich et al., 2016). In our study, sodium benzoate served as a representative of polar, aromatic contaminants (e.g., pesticides) as its physicochemical properties are similar to those of 2,4-D, dicamba or fluroxypyr (Dechesne et al., 2010a).

A heterogeneous distribution of the cells concentrated at the inoculation point clearly limited bacterial population growth and biodegradation efficiency compared to the situation with a homogeneous distribution of bacterial cells in liquid cultures. This is in line with studies on 2,4-D biodegradation in soil columns showing that biodegradation is most efficient if degraders are uniformly dispersed (Pallud et al., 2004; Pinheiro et al., 2015). By systematically decreasing the heterogeneity of the spatial degrader distribution, Dechesne et al. (2010a) also found a clear improvement of benzoate mineralization. Here, we developed an approach to estimate the impact of spatial heterogeneity on biodegradation by assessing also the performance for a quasi-optimal distribution of degraders. Thus, we found in our experiments that biodegradation performance was at less than 19% of the quasi-optimal performance if diffusion was the sole spatial process that could potentially counteract the disadvantages caused by the spatial separation between bacteria and substrate. This observation emphasizes the big impact of small-scale spatial heterogeneities for contaminant biodegradation.

### Effects of the spatial degrader distribution on the response to varying osmotic potentials

We induced osmotic stress down to ΔΨ_o_ = −1.5 MPa by adding different concentrations of sodium chloride to our experimental system, comparable to other studies (Holden et al., 1997; Chang et al., 2007). The lowest value was chosen because of its environmental relevance in representing permanent wilting point conditions for many agronomic plants in soil (Harris, 1981). In the homogeneous liquid culture system, biodegradation was reduced at ΔΨ_o_ = −1 MPa probably as a result of the reduced population growth rates and extended lag times observed in the growth kinetic experiments (Figures 2A,B). Furthermore, a homogeneous cell distribution causes a high effective exposure of the bacteria to the osmotic stress across the microcosm area. In order to resist exposure to high concentrations of salts, bacteria have evolved stress tolerance mechanisms like the accumulation of osmolytes at concentrations that are proportional to the osmolarity of the medium (Csonka, 1989; Oren, 2001). However, synthesizing osmolytes requires large amounts of energy and thus poses a significant metabolic burden for microorganisms (Oren, 1999). As a consequence, less energy is available for growth explaining the delayed growth as well as the lowered final biomass yields observed with decreasing ΔΨ_o_.

In the scenario allowing only for substrate diffusion (*D*_*dif*_), the changing osmotic potentials had only a minor impact on population growth and biodegradation probably because the diffusion limitation is the major controlling factor masking the physiological limitations imposed by the osmotic stress. However, we assume that the restricted dispersal of the cells also leads to an accumulation in the inoculation point and thus shields the bacteria from the osmotic stress. This is in line with different biofilm studies reporting an increased resistance of aggregated bacteria compared to planktonic states also for osmotic stress (Wai et al., 1998).

Several studies have demonstrated that osmotic stress may affect soil microorganisms by reducing their biomass (Tripathi et al., 2006), amino acid uptake and protein synthesis (Norbeck and Blomberg, 1998), and respiration (Gennari et al., 2007). In addition, also serious consequences for the provision of ecosystem services were reported (Stark and Firestone, 1995). Following up on this, we could show that not only the spatial heterogeneity of the degraders but also their response to osmotic stress (i.e., to a decrease of ΔΨ_o_) has to be taken into account for the natural attenuation capacity of ecosystems.

### Effects of bacterial dispersal at different osmotic potentials

In the present study, bacterial movement through the agar matrix and along the dispersal networks was found to counteract the disadvantage due to spatial degrader heterogeneity at different osmotic potentials. The counteraction ability was higher in case of glass fiber networks, which had been shown to accelerate bacterial dispersal processes earlier (Banitz et al., 2011a; Worrich et al., 2016). Glass fibers were used to simulate hyphae surrounded by liquid films (Banitz et al., 2011b; Pion et al., 2013a) and to exclude effects of hyphal activities on bacterial growth and nutrition (Furuno et al., 2012; Banitz et al., 2013; Pion et al., 2013b; Schamfuß et al., 2013).

In this study we used the flagellated bacterium *P. putida* KT2440, which can disperse by swimming motility (Dechesne et al., 2010b). We used 0.3% agar in the experiments as this concentration is supposed to facilitate bacterial swimming through water filled channels in the agar matrix (Rashid and Kornberg, 2000). In addition, the movement of bacteria in the liquid films along fungal mycelia was shown to be enabled by flagella, as non-motile bacteria were not dispersed. It remains unclear whether this movement is associated to swimming or swarming motility (Kohlmeier et al., 2005). However, we never observed any cell movement on top of 0.5% agar plates probably because swarming in *P. putida* KT2440 relies on short pili, which are only expressed under specific conditions (Matilla et al., 2007). As the mechanisms underlying bacterial movement in our microcosms were not explicitly studied we referred to it more generally as dispersal.

Bacterial dispersal is considered a key factor for efficient biodegradation in soil (Harms and Wick, 2006; Banitz et al., 2011b) and the advantageous effects were reported several times (Wick et al., 2007; Dechesne et al., 2010a; Worrich et al., 2016). Here, we could show that bacterial dispersal is able to counteract the disadvantages caused by spatial degrader heterogeneity in our microcosm setup. However, at lowered ΔΨ_o_, the benefit of bacterial dispersal in absence of dispersal networks vanished as a consequence of the osmotic stress which was shown to reduce dispersal of the bacterial population (cf. Supplementary Figure 3; Worrich et al., 2016). Bacterial dispersal is a result of growth, passive transport and motility. However, in previous experiments with a non-motile isogenic mutant of *P. putida* KT2440, we observed that growth and passive transport contributed only marginally to dispersal in the microcosms in absence of osmotic stress (Supplementary Videos 1, 2). Thus, it is likely that the reduced colony expansion observed in the experiments at lowered osmotic potentials is caused particularly by a restriction of bacterial motility. The high metabolic costs associated with the survival at low ΔΨ_o_ probably led to a downregulation of motility genes to avoid further energetic disadvantages. Indeed, reduced expression of structural genes involved in flagellum synthesis has been observed for *Pseudomonas, Bacillus*, and *Enterobacter* strains under osmotic stress conditions (Soutourina et al., 2001; Kristoffersen et al., 2007). The minor effects shown for the scenario with the immobilized cells (*D*_*dif*_) may thus further be associated with a higher energy status of the cells due to the repression of flagellum-synthesis under conditions leading to immobilization (high agar concentration in our experiments) similar to what was postulated for non-flagellated mutants of *P. putida* KT2440 showing a higher resistance to oxidative stress than the flagellated cells (Martinez-Garcia et al., 2014). At ΔΨ_o_ = −1.5 MPa, we observed bacterial dispersal exerting negative effects on benzoate biodegradation (Figure 3E). Recently, it was found that the colonization of stress-affected environments requires a critical population density in order to maintain activity in an antibiotic landscape (Hol et al., 2016). Possibly, this was the case also in our system for low ΔΨ_o_ and the primary colonizers leaving the inoculation site were not able to establish, thus reducing the overall activity in the system.

The network-mediated dispersal benefit also gradually decreased with decreasing ΔΨ_o_. However, the presence of dispersal networks always led to improvements of bacterial population growth and biodegradation performance also at low ΔΨ_o_. We hypothesize that the presence of dispersal networks creates a trade-off with a more even bacterial coverage leading to increased substrate access, but simultaneously causing a higher effective exposure to the osmotic stress. Nevertheless, it seems that the network-mediated benefits for bacteria could compensate the energetic costs of dispersal and hence prevent the downregulation of motility genes. Probably, the potential accumulation of bacteria along the network may decrease the exposure to osmotic stress and helps to exceed the critical density threshold needed for establishment under lowered ΔΨ_o_.

### Importance of spatial processes at different osmotic potentials

Bacterial dispersal ability was identified to be crucial for the fast removal of the benzoate in our system. Although diffusion could partially secure biodegradation under the compounded effects of spatial degrader heterogeneity and varying ΔΨ_o_, bacterial motility and especially the network-mediated dispersal led to considerably higher performances (Figure 4B). At low ΔΨ_o_, not the spatial arrangement of the degrader population but rather the insufficient growth caused by the salt is the bottleneck for biodegradation as shown by the growth kinetics experiment as well as by the analysis of the homogeneous reference scenario *D*_*hom*_ at the different ΔΨ_o_. Growth itself was considered as a non-spatial process as it was shown not to account for any significant changes in bacterial spatial dynamics (Pallud et al., 2004).

The influence of many soil physicochemical parameters has been measured in order to assess controlling factors determining biodegradation efficiency (Dechesne et al., 2014). However, only a few covariates have been found which represent good predictors for biodegradation activity including pH (Rodriguez-Cruz et al., 2006; Hussain et al., 2013) and moisture (Cruz et al., 2008; Monard et al., 2012). Soil pH was shown to particularly affect bacterial growth (Bååth and Arnebrant, 1994) whereas soil moisture was shown to particularly affect bacterial motility (Dechesne et al., 2010b). Therefore, as the osmotic potential influences both bacterial growth and dispersal its potential role in determining the spatial distributions and activity should not be neglected.

### Relevance for field conditions

Land degradation by salts is a major threat in arid and semi-arid regions of the world, which is mainly caused by low rainfall and high evapotranspiration (Rengasamy, 2006). Furthermore, this problem is anticipated to worsen in future due to irrigation and clearing of the native vegetation especially in agricultural lands (Pannell and Ewing, 2006; Rengasamy, 2006). However, the steadily increasing global population necessitates land use changes toward agriculture, which will at the same time also increase the amount of contaminants (primarily pesticides) applied to the soil. Given the fact that soil is characterized by a high spatial heterogeneity of degrading microorganisms, it is necessary to assess how this heterogeneity in combination with increasing salinization will affect contaminant biodegradation. Indeed, this is a rather complex question as the spatial distribution of degrading organisms in the field is not known a priori and, moreover, difficult to assess and control in replicated, comparative experiments (Dechesne et al., 2014). Therefore, we used microcosm experiments, which allow for defined changes of the relevant conditions but do not incorporate the full complexity of the soil environment. These simplifications, however, constitute a prerequisite to better understand the importance of single aspects in natural systems (Drake et al., 1996). Nonetheless, transferring our findings to natural soil systems, one needs to acknowledge that other factors may also influence the processes elucidated in this study. Especially the matric potential, which is the second major determinant of the water potential in terrestrial habitats, is known to adversely affect bacterial dispersal and substrate diffusion processes in soil (Harris, 1981; Dechesne et al., 2008). This was addressed by several studies without consideration of the osmotic potential. However, future studies should focus on the combined effects of osmotic and matric potential, in particular if considering that decreasing water amounts in soils always result in higher salt concentrations (Chowdhury et al., 2011).

Our work demonstrates that it is important to consider the spatial distribution of degraders as a driving factor for biodegradation. Hence, bacterial dispersal and especially bacterial dispersal along fungal mycelia should be facilitated by avoiding (i) desiccation and high evapotranspiration causing low osmotic potentials, and (ii) the energy-intensive mechanical mixing of soil. The latter prevents the mycelium development of fungi (Lamar et al., 1994), which we demonstrated to counteract the disadvantages of a heterogeneous bacterial distribution. Taking into account a length of up to 10^4^ m of hyphae per g of soil (Ritz and Young, 2004) and a high tolerance toward low water activities and matric potentials, fungi seem to be a suitable and energy-efficient but yet unexploited alternative to conventional remediation approaches (Harms et al., 2011).

## Author contributions

AW designed the research and performed the laboratory work. AW, SK, TB, FC, and MT analyzed the data. SK, TB, FC, KF, MT, HH, AM, LW, and MK provided consultation for the work. AW wrote the manuscript. All authors contributed significantly to the preparation of the manuscript and approve its submission.

### Conflict of interest statement

The authors declare that the research was conducted in the absence of any commercial or financial relationships that could be construed as a potential conflict of interest.
