# Supplementary material for: Bacterial Dispersal Promotes Biodegradation in Heterogeneous Systems Exposed to Osmotic Stress
Source: Front Microbiol. 2016 Aug 3;7:1214. doi: 10.3389/fmicb.2016.01214 (PMC4971104; doi:10.3389/fmicb.2016.01214)
Supplement: Supplementary file 3 [file Image1.PDF]

## *Supplementary Material*

# Bacterial dispersal promotes biodegradation in heterogeneous systems exposed to osmotic stress

**Anja Worrich[1,2], Sara König[1,3], Thomas Banitz[3], Florian Centler[1], Karin Frank[3,4,5], Martin Thullner[1], Hauke Harms[1,4], Anja Miltner[2], Lukas Y Wick[1*] and Matthias Kästner[2]**

**Correspondence:** Lukas Y. Wick; lukas.wick@ufz.de

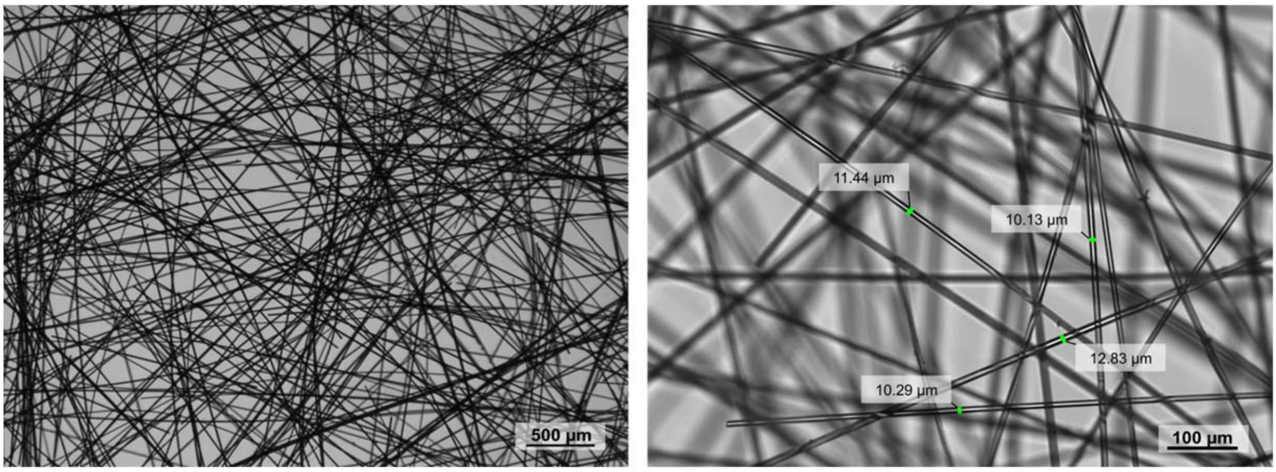


**Supplementary Figure 1.** Micrograph of the glass fiber network used to simulate fungal mycelia. Green lines indicate the position used to measure fiber diameters.

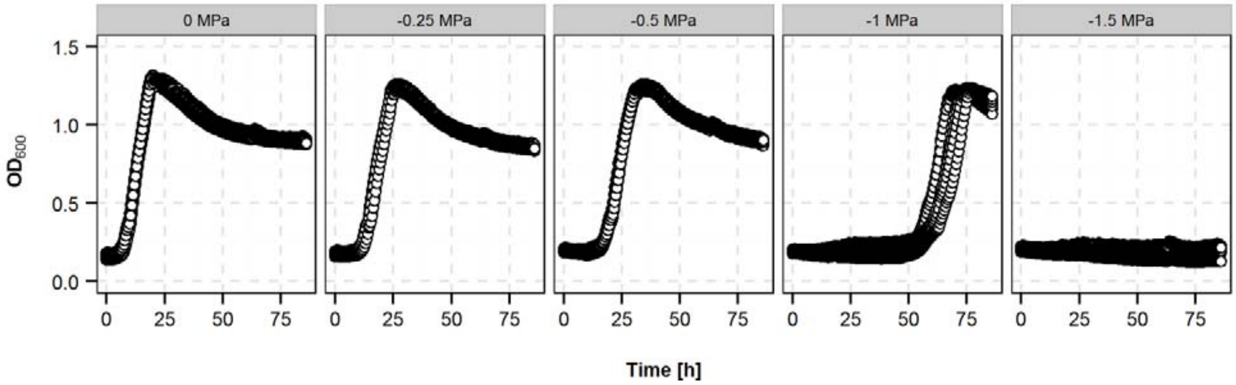


**Supplementary Figure 2.** Growth curves of *Pseudomonas putida* KT2440 on 50 mM sodium benzoate at different osmotic potentials (indicated by the subplot label) adjusted by the addition of sodium chloride. Growth was measured spectrophotometrically at a wavelength of 600 nm every 30 min. Obtained growth curves were used to derive maximum specific growth rates, lag times and maximum biomass.

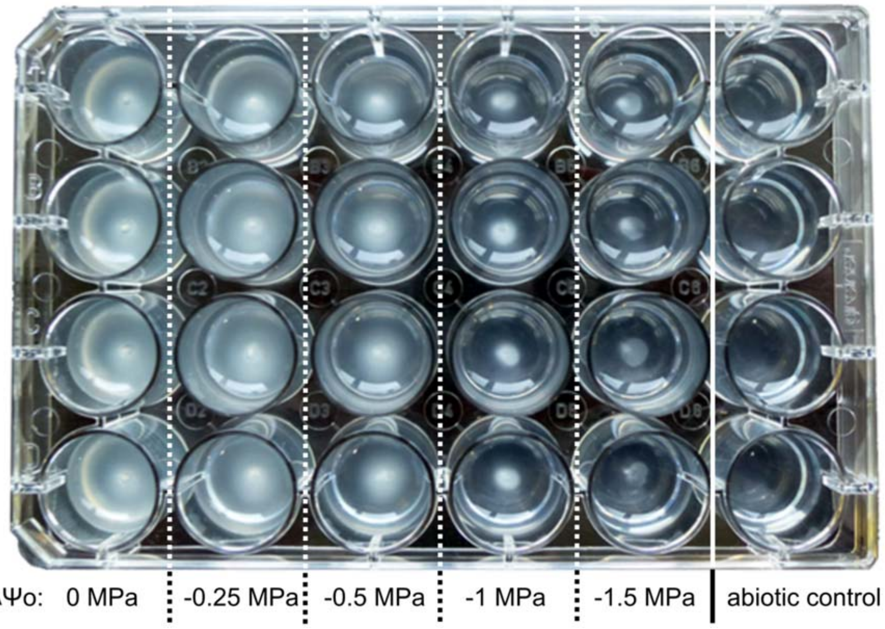


**Supplementary Figure 3.** Microcosm colonization by *P.putida* KT2440 after 30 h of incubation in the $D_{dis}$ scenario at different osmotic potentials.

**Supplementary Video 1:** Time-lapse video showing the population dispersal of the flagellated wild type *P.putida* KT2440 in $D_{dis}$ scenario at $\Delta\Psi_o = 0$ MPa. The video was constructed from 49 images taken at 30 min intervals.

**Supplementary Video 2:** Time-lapse video showing the population dispersal of a nonflagellated isogenic mutant of *P.putida* KT2440 in $D_{dis}$ scenario at $\Delta\Psi_o = 0$ MPa. The video was constructed from 49 images taken at 30 min intervals.
